# The complete chloroplast genome sequence and phylogenetic analysis of *Disporum viridescens (*Liliaceae)

**DOI:** 10.1080/23802359.2022.2073849

**Published:** 2022-06-10

**Authors:** Mingyan Ye, Jiaqi Lang, Fuqiang Yin, Ming Liu

**Affiliations:** a College of Biological and Food Engineering, Chongqing Three Gorges University, Chongqing, China; b The Chongqing Engineering Laboratory for Green Cultivation and Deep Processing of the Three Gorges Reservoir Area’s Medicinal Herbs, Chongqing, China

**Keywords:** *Disporum viridescens*, chloroplast genome, phylogeny

## Abstract

*Disporum viridescens* is a medicinal plant in three provinces of Northeast China. In this paper, we report the characteristics of the complete chloroplast genome (CP) of *Disporum viridescens.* We discuss mainly the phylogenetic relationship between this species and its relatives. The length of its sequence was 156,645 bp, with a total GC content of 37.6% A large single-copy region (LSC) of 85,103 bp, a small single-copy region (SSC) of 17,964 bp, and a pair of inverted repeat (IR) regions of 26,789 bp were detected in this study. The complete chloroplast genome sequence contained 127 genes, including 81 encoding, 38 transfer RNA (tRNA), and 8 ribosomal RNA (rRNA) genes. Our phylogenetic analysis results showed that *Disporum viridescens* is closely phylogenetically related to *Disporum cantoniense* of the family Colchicaceae.

*Disporum viridescens*, which was first described by Carl Johann (Ivanovič), Maximowicz in 1859 (Maxim. 1859). Liliaceae is a large and complex family (Zhou et al. 2010), distributed in Guizhou, Yunnan province in China and other parts of the world such as Japan, Europe. Seventeen *Disporum* species grow in China, of which 10 are endemic to the country (Zhang et al. 2021). *Disporum viridescens* is a medicinal plant belonging to the genus of *Disporum* in the Liliaceae family. The chemical ingredients are mainly whole herbs containing tannins and saponins (DAI). This plant has beneficial medicinal effects, including clearing of the lungs, cough relief, and spleen and stomach strengthening (Wang 1978). In China, it is mainly distributed in forests, hillsides, and grasslands (Gao 2010) in Heilongjiang, Jilin, and Liaoning provinces. Yabe collected *Disporum viridescens* for the first time in Fengtian City (now referring to Shenyang City), Liaoning Province on August 25, 1909. However, few studies have been conducted on the molecular biology of this medicinal plant. Therefore, we performed an investigation on the chloroplast genome sequence of *Disporum viridescens*. We revealed its internal relationship with other plants in Angiospermae, which is conducive to further research on the molecular and phylogenetic characteristics of *Disporum viridescens*.

On 30 May 2021, fresh plant specimens of *Disporum viridescens* were collected in Dandong City, Liaoning Province (40°03′26.5″N, 124°10′28.7″E). The specimens were identified by Nong Zhou and placed at the herbarium of Chongqing

Three Gorges University (https://www.sanxiau.edu.cn, Nong Zhou and erhaizn@126.com) under voucher number No. ZN20210630. Total DNA was isolated from dried root materials, and second-generation sequencing was performed using Illumina HiSeq 2500 platform (Novogene, Tianjin, China) using a previously reported modified CTAB method (Yang et al. 2014). From high-throughput sequencing was about 2.56 Gb of raw data. To eliminate the redundant data, the original reads were filtered by Trimmomatic v.0.32 software with default parameters (Bolger et al. 2014). Then, the obtained clean reads were assembled into circular contigs using GetOrganelle (Jin et al. 2020) using *Disporum viridescens* as a reference. Afterwards, the cpDNA was annotated by the Dual Organellar GenoMe Annotator CpGAVAS2 (Shi et al. 2019). Finally, the assembled chloroplast genome was submitted to the GenBank under the accession number OL840058.

The whole genome of *Disporum viridescens* has a typical tetragonal structure. The length of the sequence was 156,645 bp, with a total GC content of 37.6%. We identified a large single-copy region (LSC) of 85,103 bp, a small single-copy region (SSC) of 17,964 bp, and a pair of inverted repeat (IR) regions of 26,789 bp. The complete chloroplast genome sequence was found to contain 127 genes, including 81 encoding, 38 transfer RNA, and 8 ribosomal RNA genes.

To investigate the taxonomic status, CP genome sequences of 31 species were downloaded from the NCBI database for phylogenetic analysis. After using MAFFT V.7.427 software (Katoh and Standley 2013) for aligning, a maximum-likelihood (ML) tree was constructed by MEGA v.7.0 (Kumar et al. 2016) with 1000 bootstrap replicates. Two Iridaceae species (*Iris domestica* (MT001880) and *Iris tectorum* (MT103435)) were utilized as outgroups. The results showed that *Disporum cantoniense* was closely related to *Disporum viridescens* (Figure 1).

**Figure 1.**
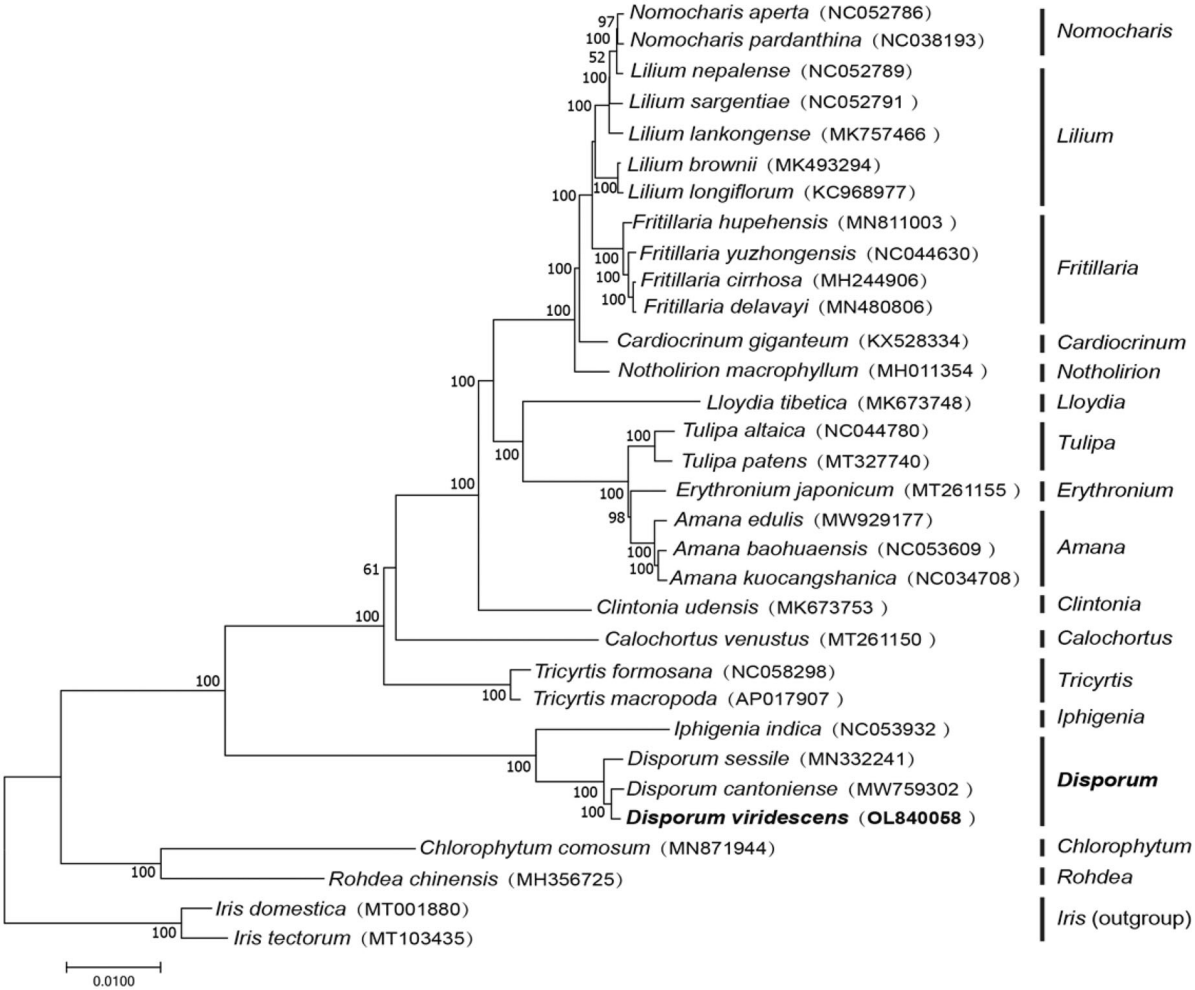
ML phylogenetic trees of 32 species were constructed using the complete chloroplast genomes of two Iridaceae species (*Iris domestica* (MT001880) and *Iris tectorum* (MT103435)) as outgroups.

## Ethical approval

No specific permissions were needed to perform this research as *Disporum viridescens* is not a protected plant, and no damage could be caused to its population.

## Author contributions

Ming Liu was mainly responsible for the design of the experiment and approved the final version of the paper; Mingyan Ye was mainly responsible for the writing and revising of the paper, Jiaqi Lang and Fuqiang Yin participated in the sample assembly and annotation work, Mingyan Ye and Ming Liu analyzed and explained the data; All authors are accountable for all aspects of the work.

## Data Availability

The data of this study are available in GenBank of NCBI at (https://www.ncbi.nlm.nih.gov) under accession no. OL840058. The associated BioProject, SRA, and BioSample numbers are PRJNA801161, SRR17798981, and SAMN25349808, respectively.
